# Investigation of wind load behavior on hip roof structures subjected to Tornado-induced flow

**DOI:** 10.1371/journal.pone.0335098

**Published:** 2026-09-03

**Authors:** Xiaolong Hao, Yanzhen Jia, Xueyan Wang, Wenlong Cao, Zhibo Zhang

**Affiliations:** 1 Hebei Provincial Seasonal Frozen Area Highway Service Safety and Early Warning Technology Innovation Center, Shijiazhuang, Hebei, China; 2 Department of Road and Bridge Engineering, Hebei Jiaotong Vocational and Technical College, Shijiazhuang, Hebei, China; 3 School of Economics and Management, Shijiazhuang University, Shijiazhuang, Hebei, China; 4 School of Civil Engineering, Shijiazhuang Tiedao University, Shijiazhuang, Hebei, China; Universiti Teknologi Malaysia - Main Campus Skudai: Universiti Teknologi Malaysia, MALAYSIA

## Abstract

This study investigates the effects of ground roughness, building orientation, swirl ratio, and radial position on the wind loads of hip roof buildings under tornado conditions. The simulation results (CFD) presented herein were compared with validated simulations, existing experimental data, and field measurements to validate the accuracy of the modelling approach. The most unfavorable vortex ratio affecting the hipped roof was identified through an analysis of the wind pressure coefficient under typical vortex ratios. The wind load characteristics at specific roof locations were examined by comparing radial distances. The influence of orientation angle on turbulent structures and wind pressure distribution within the wind field was investigated through comparative analysis of different aspects. This study investigates the influence of ground roughness on wind pressure and mean aerodynamic force over hipped roofs through computational analysis. Results indicate that increased surface roughness reduces the proportion of upward airflow, causing wind field characteristics to approach those of smooth surfaces. When the building orientation is 90°, the maximum negative pressure occurs at the leeward ridge junction due to minimal airflow separation and windward area. At the maximum core radius, airflow directly impacts the windward surface, inducing flow separation and vortex formation, which generates substantial vortices on the roof’s leeward side. These findings provide quantifiable insights into the near-ground tornado-structure interaction, offering valuable guidance for optimizing design codes and enhancing the resilience of low-rise buildings in tornado-prone regions.

## 1. Introduction

Tornadoes frequently occur in suburban, rural, and forested regions, where low-rise gabled and hip roof buildings are predominant [1,2]. There is substantially less current study on hip roof subjected to tornado activity than there is on gabled roofs. Therefore, it is necessary to investigate the wind load characteristics of tornadoes on hip roof to provide reference for disaster mitigation and prevention. The tornado is a strong vortex phenomenon in the atmosphere, a kind of local, small-scale, sudden strong convective weather. Moreover, the tornado is convective air movements that produce strong, tiny air vortices under highly unstable meteorological conditions. In March 2023, a series of devastating EF-4 tornadoes swept across the southern United States, causing catastrophic damage in Mississippi. The storms resulted in 26 fatalities and destroyed over 90% of structures in several affected towns, leaving entire communities nearly completely leveled [3]. Even modern building complexes remain highly vulnerable to the most powerful tornadoes, underscoring the urgency of specialised tornado-resistant research. Currently, building wind resistance design codes in many regions primarily address atmospheric boundary layer winds, with design wind speeds typically based on a 50-year recurrence interval standard, generally set at 25-30m/s [4]. However, even an EF-2 tornado’s wind speeds far exceed conventional building design standards. This significant gap between design standards and actual extreme events means a substantial number of existing buildings face extremely high risks during tornado strikes.

The internal pressure and the local net wind force on the roof of a building model with a stationary wind field are significantly influenced by the ground roughness. As roughness increases, the local net wind force at the top intensifies, while the internal pressure on the building model decreases accordingly. Building roofs experience progressively greater suction due to intensified core updrafts. In the presence of roughness, the disparity in pressure coefficients between the front and rear boundaries of the structure diminishes [5,6]. The,influence of ground roughness on external pressure is confined to a range of 3–5 times the height of the roughness unit [4].

Not only does ground roughness exert a significant influence on the tornado wind field, but the distance between the tornado’s centre and the model, along with the azimuth of the buildings, also markedly affects the turbulent structure surrounding the low-rise building model. The separated flow’s vortex structure and local pressure distribution are altered by the tornado’s wind field’s curvature. The curvature of the horizontal flow field surrounding the building determines the magnitude of azimuthal displacement. The larger the required azimuthal displacement, the more curvature there is in the circulation [7,8]. When the building model is located at the tornado’s core radius, the peak suction pressure coefficient occurs near the windward eave leading edge. Notably, the mean lift force acting on the leeward portion of the roof is significantly larger than that on the windward roof portion [9,10].

Furthermore, the wind pressure and average aerodynamic forces acting upon the surfaces of low-rise buildings provide a reference basis for wind-resistant design. The complex wind field characteristics within tornado vortex cores—particularly the presence of secondary vortices and vertical wind components—are the primary cause of reduced simulation accuracy for tornadoes [11]. Over extended durations, peak pressures may increase by 1.1 to 1.4 times. The vortex core or its immediate vicinity experiences the highest pressure peaks [12]. The influence of building size effects and vortex-dynamic structural effects on the pressure coefficient of buildings within tornadoes. Significant discrepancies exist between pressures in regions of pronounced flow separation and the overall pressure distribution across building surfaces. Tornado-induced pressures typically exceed equivalent linear wind pressures by 13% [3,13]. The enhanced lift on gable roofs under tornado conditions is primarily attributed to ground roughness-induced reduction in vortex core radius, which alters the swirl ratio and intensifies vertical updrafts [14].

Finally, the vortex ratio, translational velocity, and the distance between the structure and the tornado’s average path are also significant factors influencing the wind field. The location of a tornado exerts a significant influence on its dynamic pressure characteristics. Pressure behavior is primarily governed by vortex-induced pressure reduction. Positive pressure distributions occur across multiple surfaces of porous structures subjected to tornado flow fields [15,16]. Surface pressure characteristics are strongly influenced by the tornado’s translational trajectory, speed, and direction relative to the structure, with abrupt changes in both magnitude and direction of aerodynamic forces occurring as the tornado core approaches [17–20]. The intensity and distribution of wind forces around architectural models exhibit a significant correlation with the distance from the tornado vortex. Localized pressure on building roofs is closely related to roof angle. As tornado translational velocity increases, the mean drag force acting on low-rise buildings consequently intensifies [1,21]. These complex flow phenomena, including strong reverse pressure gradients and flow separation, pose significant challenges for numerical simulation. Standard turbulence models struggle to accurately capture flow details within the separated shear layer and near-wall regions. Consequently, this study employs the Shear Stress Transport (SST) k−ω turbulence model. This model combines the strengths of the k−ω model in near-wall regions with those of the k−ε model in the far-field. It resolves the complex fluid physics arising from the interaction between the four-sloped roof geometry and the tornado. The aforementioned literature has examined low-rise buildings from individual aspects such as ground roughness, directional angle, and aerodynamic forces. Roof configurations primarily encompass flat roofs and gable roofs. While valuable aerodynamic databases for hip roof buildings under conventional atmospheric boundary layer (ABL) winds have been established [22–24], there remains a critical lack of comprehensive, systematic research investigating hip roof wind load characteristics under tornado conditions through the integrated analysis of ground roughness, swirl ratio, building orientation, aerodynamic forces, and radial position relative to the tornado core.

To investigate the interaction mechanism between tornado wind fields and hipped roofs. This paper employs the SST k−ω turbulence model to analyse the wind load characteristics of a hipped roof subjected to tornado forces. This study will compare the simulation results of the flat roof model to verify the correctness of the established tornado field and structural coupling simulation. It identifies the most unfavorable vortex ratio, the most unfavorable rotation angle, and the maximum affected radial distance. The locations on the hipped roof experiencing the highest wind pressure and aerodynamic forces during a tornado are specified, providing a reference basis for the wind-resistant design of hipped roof structures.

## 2. Numerical simulation and reliability verification of tornadoes

### 2.1. Turbulence model and control equations

In this paper, transient solutions are obtained using the SST *k-ω* turbulence model. The SST *k-ω* turbulence model is a hybrid model developed by Menter [25], which belongs to the near wall function. The SST *k-ω* turbulence model retains the standard *k-ω* near the wall, while the *k-ε* model is applied far away from the wall. Compared with the two turbulence models, the *k-ε* model is only limited to the turbulent boundary layer pressure phase, and its wall function is difficult to make up for the gap between the calculation model and the actual physical phenomenon in the correction of the boundary layer. However, the SST *k-ω* turbulence model makes up for these shortcomings. On the one hand, the SST *k-ω* model can adapt to various physical phenomena of pressure gradient changes. On the other hand, the SST *k-ω* model can be applied to the viscous inner layer, which can accurately simulate the phenomenon of the boundary layer. Kharicha [26] also used the SST *k-ω* model for transient calculation to obtain transient results, so as to study the characteristics of tornado wind field. The transverse dissipative derivative term is also added to this turbulence model for the turbulent kinetic energy and dissipation rate equations:


$$\begin{array}{c} \frac{\partial }{\partial t}\left(\rho k\right)+\frac{\partial }{\partial x_{i}}\left(\rho ku_{i}\right)\text{\textbackslash{}hspace\{0.33em}\}=\text{\textbackslash{}hspace\{0.33em}\}\frac{\partial }{\partial x_{i}}\left(\Gamma_{k}\frac{\partial k}{\partial x_{i}}\right)+G_{k}-Y_{k}+S_{k}\text{\textbackslash{}hspace\{1em}\;\;\;\;\;\;\;\;\} \end{array}$$ (1)

$$\begin{array}{c} \frac{\partial }{\partial t}\left(\rho \text{\textbackslash{}hspace\{0.33em}\}\omega \right)+\frac{\partial }{\partial x_{i}}\left(\rho \text{\textbackslash{}hspace\{0.33em}\}\omega u_{i}\right)=\frac{\partial }{\partial x_{j}}\left(\Gamma_{\omega }\frac{\partial \omega }{\partial x_{j}}\right)+G_{\omega }-Y_{\omega }+D_{\omega }+S_{\omega }\text{\textbackslash{}hspace\{1em}\;\;\;\;\;\;\} \end{array}$$ (2)

The turbulent kinetic energy is k, ω is the dissipation rate, and the air density is ρ.where $G_{k}$ and $G_{\omega }$ are the mean velocity gradients generated by k and ω, respectively. Valid diffusion terms for k and ω are $\Gamma_{k}$ and $\Gamma_{\omega }$, respectively. $Y_{K}$ and $Y_{\omega }$ are the diffusion terms of k and ω,respectively. $D_{\omega }$ is the orthogonal divergence term. What is more, $S_{k}$ and $S_{\omega }$ are user-defined terms.

### 2.2. Tornado generating device and Geometric model

**Fig 1:**
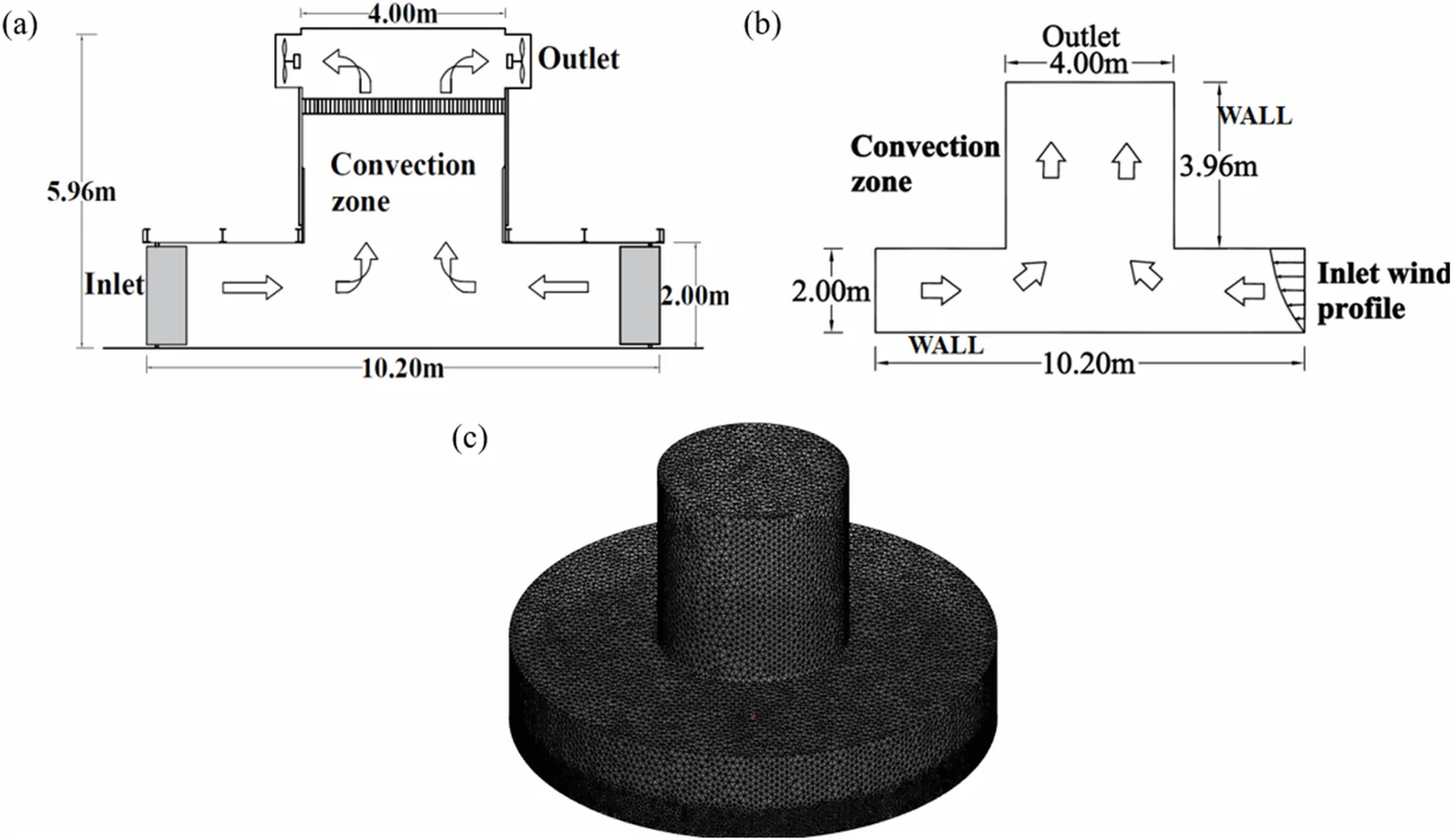
Tornado generating device and grid division, (a) VorTECH model dimensions [29] (adapted from Zuo et al., 2021), (b) tornado numerical model dimensions, (c) tornado generator tetrahedral meshing.

**Fig 2:**
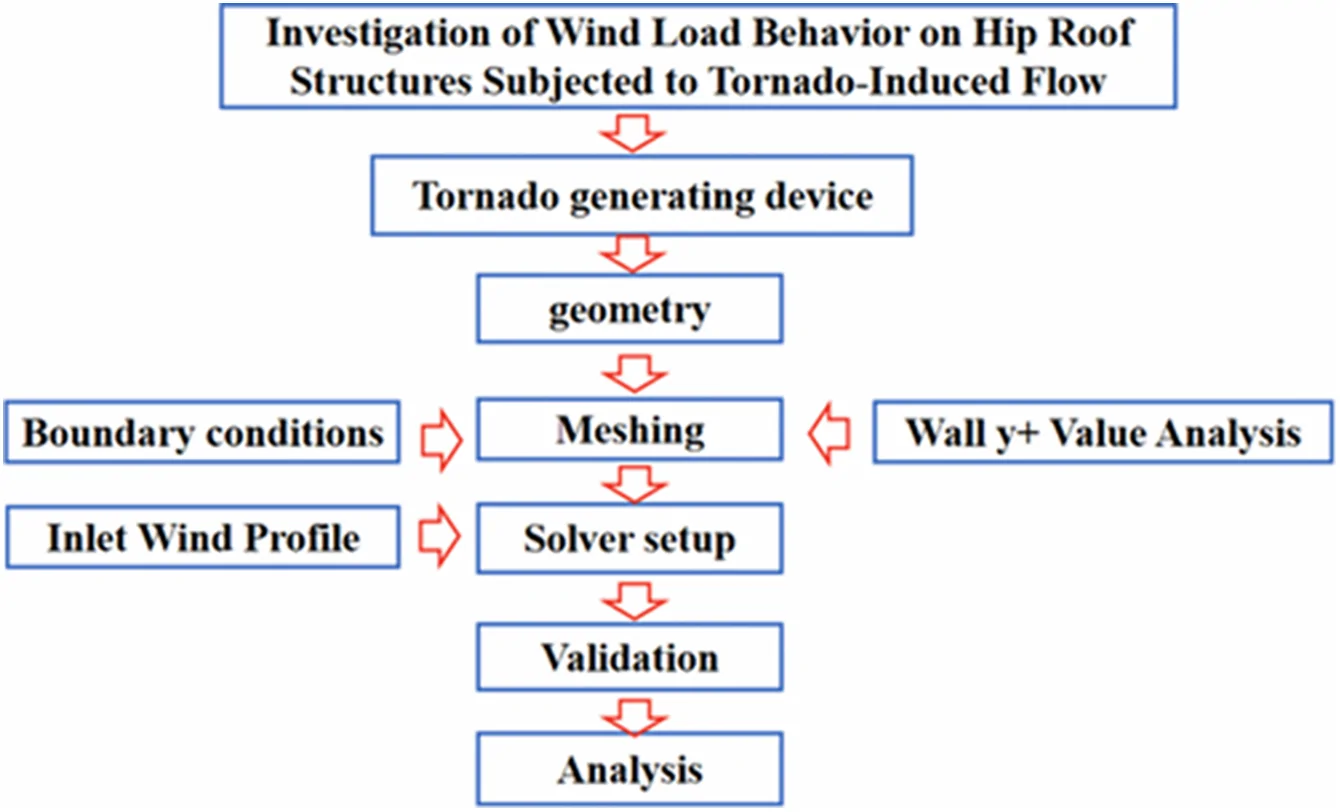
Tornado CFD workflow.

Tornado wind field and building model interactions were primarily explored through tornado generation devices. The Ward type [27] tornado generating device was proposed and built by Ward in 1972. The VorTECH [28], which is one of the Ward-type tornado generators proposed by Texas Tech University (TTU), is utilized in this study. Tornado generating device and grid division are shown in Fig 1. ICEM software was employed for modelling purposes, and simulations were conducted using Fluent. Fig 2 illustrates the tornado CFD workflow.

**Fig 3:**
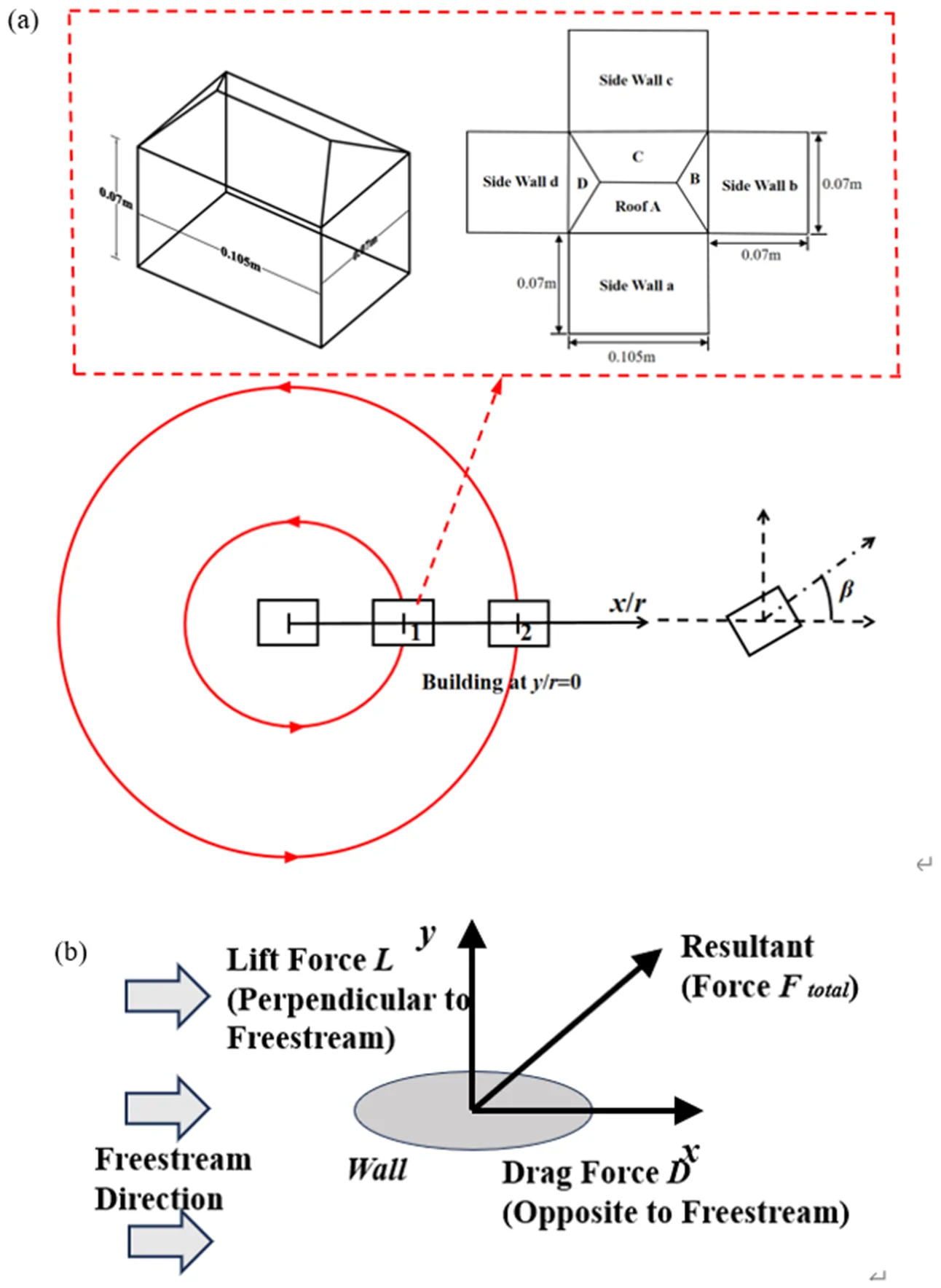
Building size position and direction of incoming flow and aerodynamic force. (a) Size and location of the hip roof building (b) Incoming flow and aerodynamic direction.

The building model and the tornado generator are modeled at a scale of 1:100. We used the Ward type tornado generator proposed by Texas Tech University (TTU). The experimental model in the tornado generator and simulator we used was built in the same proportion as the 1:100 model of the wind engineering research field laboratory building of Texas Tech University. Many literatures use the scale of 1:100 to explore. For example, studied low-rise building models with a scale of 1:100 based on a large-scale Ward-type tornado simulator VorTECH at Texas Tech University. The tornado wind field parameters are shown in Table 1. The size of the hip roof building is 0.105 m x 0.07 m x 0.07 m, and the slope angle of the model is 45°.The roof is divided into four partitions A, B, C and D in counterclockwise direction. The location and direction of the building dimensions of the incoming flow and aerodynamic forces are shown in Fig 3. The largest core radius is *R*_max_, and *r* is where the building model is located in the wind field. The building model is placed at different positions in the wind field with *r* = 0*R*_max_, *r* = 0.5*R*_max_, *r* = 1.0*R*_max_, *r* = 1.5*R*_max_, *r* = 2.0*R*_max_, *r* = 2.5*R*_max_ and *r* = 3.0*R*_max_. The different orientations of the building model are at 0°, 30°, 45°, 60°, and 90° at the biggest core radius.

**Table 1:** Parameters of tornado wind field.

Reference	Model Scale	*S*	*R*_max_	*V*_tmax_
Tang [30]	1:100	0.84	0.52 m	12.9 m/s
Present study1	1:100	0.18	0.2 m	7.5 m/s
Present study2	1:100	0.74	0.43 m	10 m/s

### 2.3. Boundary conditions and solution methods

A User-Defined Function (UDF) is implemented to define the inlet boundary conditions, including the tangential velocity, radial velocity, and vertical velocity profiles. The tangential velocity (*V*_*t*_) and the radial velocity (*V*_*r*_) formula of the inlet are as follows:


$$\begin{array}{c} V_{\text{t}}=U_{\text{0}}\text{\textbackslash{}hspace\{0.5em}\}{\left(\frac{Z}{Z_{\text{0}}}\right)}^{\frac{\text{1}}{n}}\text{\textbackslash{}hspace\{1em}\;\;\;\;\;\;\;\;\;\;\;\;\;\;\;\;\;\;\} \end{array}$$ (3)

$$\begin{array}{c} V_{\text{t}}=2V_{\text{r}}S\left(\frac{H_{\text{0}}}{R_{\text{0}}}\right)\text{\textbackslash{}hspace\{1em}\;\;\;\;\;\;\;\;\;\;\;\;\;\;\;\;\;\} \end{array}$$ (4)

$$\begin{array}{c} tan\theta =\frac{2SH_{\text{0}}}{R_{\text{0}}}\text{\textbackslash{}hspace\{1em}\;\;\;\;\;\;\;\;\;\;\;\;\;\;\;\;\;\;\;\;\;\;\;\} \end{array}$$ (5)

Where $V_{\text{r}}$ and $V_{\text{t}}$ are the radial and tangential velocities, respectively, and *n* is taken as 7 for the neutral stability condition. The radial velocities at the reference height are represented by the $Z_{\text{0}}$ and $U_{\text{0}}$, respectively. The inflow angle’s magnitude is represented by θ, and the swirl ratio is represented by S [29]. The tornado generating device’s inflow height and outflow region radius, respectively, are $H_{\text{o}}$ and $R_{\text{o}}$. The tornado generator are simulated on a scale of 1:100. The value of $U_{\text{0}}$ was fixed to 0.39 m/s by comparing the velocity profile at the entrance to Tang’s experimental [31] findings, where the wind field swirl ratio was determined to be *S*=0.18. The boundary conditions and wind field parameters are shown in Table 2, respectively. Pressure-outlet is the static pressure of a given flow outlet. The pressure outlet boundary can be used to provide better convergence when there is backflow at the outlet during the iteration process. The ‘Wall’ boundary condition is used to represent the boundary between the fluid and the solid wall, and the velocity and other properties of the fluid are limited by the wall. The matching dimensionless parameters in this paper include swirl ratio, aspect ratio and ground roughness. The unmatched dimensionless parameters are only Reynolds number.

**Table 2:** Boundary conditions and wind field parameters.

Parameters	Present numerical simulation
Confluence area radius: *R*_0_	2.0 m
Height of inlet zone: *H*_0_	2.0 m
Convection zone height: *L*	3.96 m
Swirl ratio: *S*	0.18
Inlet	Velocity inlet
Outlet	Pressure outlet
Ground	Wall
Inner side	Wall
Calculation	Transient
Turbulence model	SST *k-ω*
Discrete format	Second-order upwind
Convergence precision	10^−4^
Maximum tangential wind speed: *v*_tmax_(*S*=0.18)	7.5 m/s
The radius of the inflow area	*d*
The height of the inflow area	*z*
Radius of inflow area	*r*
Tangential velocity	*V*_*t*_
Radial velocity	*V*_*r*_
Maximum core radius	*R*_max_
Maximum tangential velocity	*V*_tmax_
Air density	1.225 kg.m^-3^

### 2.4. Key parameter definitions

The radial Reynolds number is represented by $Re_{r}$, and the Reynolds number is the ratio of the inertial force to the viscous force of the fluid motion. The volume flow rate of the device in this study is 73.66 m^3^/s. The radial Reynolds number $Re_{r}$ = 7.82 × 10^5^ is obtained by calculation. It is found that when the radial Reynolds number $Re_{r}$ ≧10^5^, the influence of the tornado generator on the tornado can be ignored [32–34]. Document 26 reanalyzed Ward’s experimental data and found that the dimensionless core radius primarily depends on the swirl ratio and is insensitive to the Reynolds number. Even with Reynolds number variations spanning 10⁴ orders of magnitude, the core radius remains strongly correlated with the swirl ratio. This indicates that viscous effects in radial flow are not the dominant factor in core scale formation. The wind pressure coefficient and aerodynamic characteristics of the building model surface are examined in order to more thoroughly examine the interaction between the tornado wind field and the building model. The expressions for the resistance coefficient and lift coefficient, two common aerodynamic property factors, are as follows:


$$\begin{array}{c} Re_{r}=\frac{Q}{2\pi v}\text{\textbackslash{}hspace\{1em}\;\;\;\;\;\;\;\;\;\;\;\;\;\;\;\;\;\;\;\;\;\;\;\} \end{array}$$ (6)

$$\begin{array}{c} C_{\text{p}}=\frac{P-P_{\text{ref}}}{0.5\rho V_{\text{tmax}}^{\text{2}}}\text{\textbackslash{}hspace\{1em}\;\;\;\;\;\;\;\;\;\;\;\;\;\;\;\;\;\;\} \end{array}$$ (7)

$$\begin{array}{c} C_{Fp,i}=\frac{F_{p,i}}{0.5\rho V_{tmax}^{\text{2}}A_{i}}\text{\textbackslash{}hspace\{1em}\;\;\;\;\;\;\;\;\;\;\;\;\;\;\;\;\;\;\} \end{array}$$ (8)

$$\begin{array}{c} C_{Fw,i}=\frac{F_{w,i}}{0.5\rho V_{tmax}^{\text{2}}A_{i}}\text{\textbackslash{}hspace\{1em}\;\;\;\;\;\;\;\;\;\;\;\;\;\;\;\;\;\;\} \end{array}$$ (9)

The inlet height’s volume flow rate is represented by *Q*. The kinematic viscosity of air is 1.5 × 10^−5^ m^2^/s, denoted by the letter *v*. Where P is the measurement point wind pressure, $P_{\text{ref}}$ is the reference point static pressure, and the reference point static pressure is the mean static pressure at the inlet boundary of the computational domain. The air density is ρ. $V_{\text{tmax}}$ is the maximum value on the horizontal section of the height tangential velocity of the model obtained by the air simulator without building model. When the swirl ratio is 0.18, the value of $V_{\text{tmax}}$ is 7.5 m/s. The typical lift and drag coefficients are $C_{Fp,i}$ and $C_{Fw,i}$. $F_{pi}$ and $F_{w,i}$ are the lift and drag forces. $A_{i}$ indicates the projected area of the building model in the corresponding direction. The aerodynamic coefficient serves as the fundamental link between raw experimental data and subsequent physical analysis. Its pivotal role in subsequent analysis manifests in three aspects: (1) Quantifying and comparing flow characteristics under different parameters in a dimensionless form, such as vortex structure and pressure distribution. (2) Serving as a direct input parameter for evaluating wind load mechanisms on structures and structural failure modes. (3) Enabling parametric analysis by examining how key coefficients vary with factors such as the vorticity ratio and radial position, thereby directly revealing dominant physical interactions.

## 3. Simulation results and analysis

### 3.1. Flat roof model verification and grid independence verification

**Fig 4:**
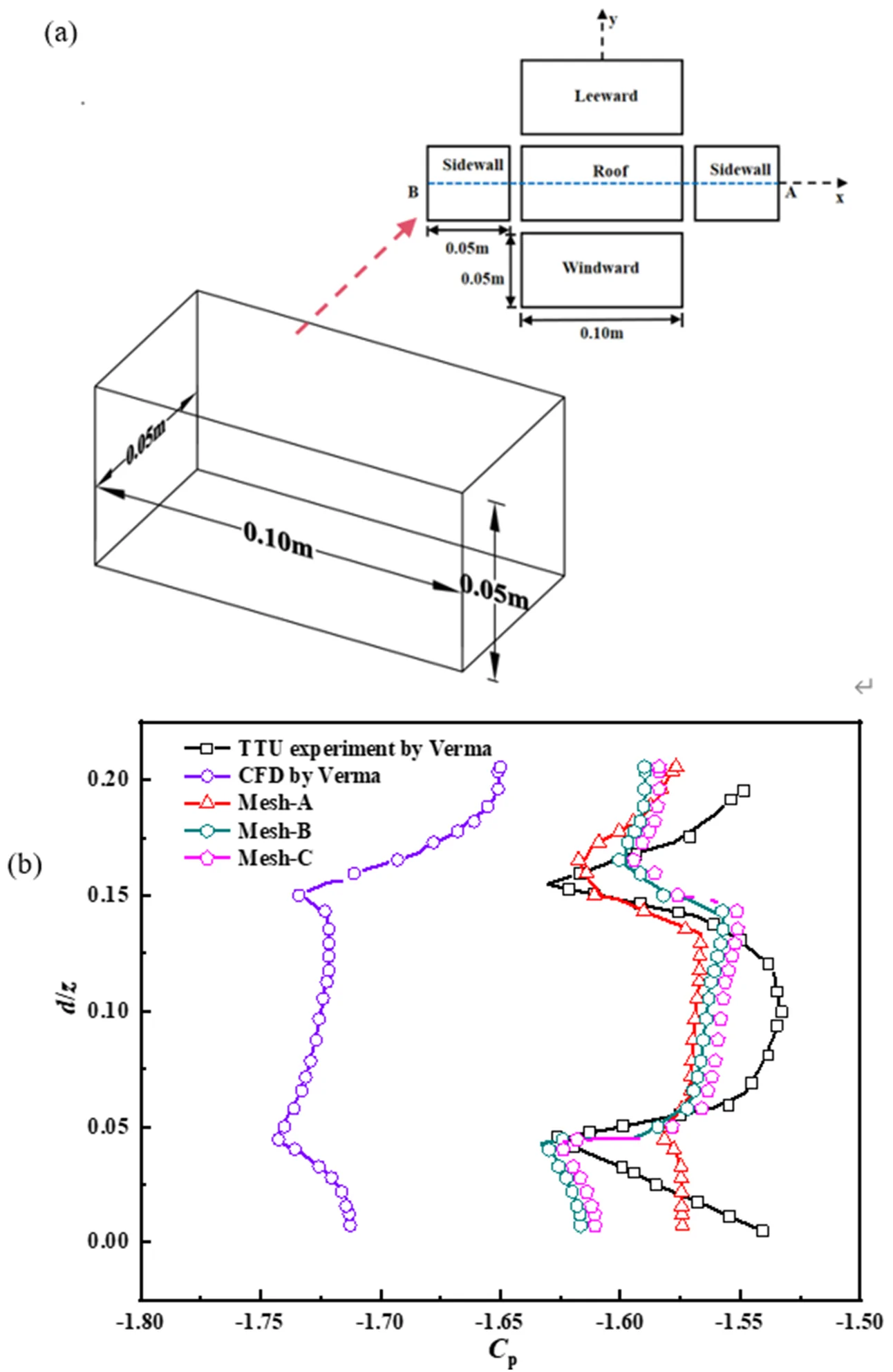
Verification of flat roof model size and wind pressure coefficient. (a) Flat roof size and expansion diagram, (b) Comparison and verification of flat roof wind pressure coefficient.

Unstructured meshing is used to guarantee the effectiveness and quality of the meshing process. The best meshing technique is determined by comparing and analyzing the simulation results of the three methods in order to confirm the grid independence. The parameters for various mesh sizes are displayed in Table 3. Fig 4 shows the size of flat roof model and the verification of wind pressure coefficient. The flat roof model is located in the core of the tornado wind field, with a size of 0.1 m x 0.05 m x 0.05 m. The scale ratio of the model is 1:100, and the center line AB of the building is the detection point position. The radius of the inflow area is *d*, and the height of the inflow area of the simulator *z*. The simulation results of grids A, B and C are consistent with the overall trend of the experimental results, especially the results of Mesh-B are closer to the experimental results [3,35], which verifies the reliability and accuracy of the simulation method again. The number of grids increases from A to B, and the simulation results are closer to the experiment. The number of grids increases from B to C, and the simulation results change little. It can be seen that the simulation effect of Mesh-B is better, so this paper adopts the division method of grid B. The average relative deviation of the simulation results between Mesh-B and Mesh-C is 0.3%. This grid convergence study, demonstrating minimal variation (0.3%) between the two finest meshes, serves as a core component of the numerical uncertainty analysis for the present CFD simulation, quantifying its sensitivity to spatial discretization. This variation stems from changes in wind field pressure caused by alterations in the tangential velocity component.

**Table 3:** Different Grids considered for Mesh Convergence Study with their mesh sizes.

Grid	y+	Total elements	Smallest size of grid	Largest size of grid
Mesh-A	10	2,201,117	0.004*h*	0.04*h*
Mesh-B	5	2,696,815	0.0018*h*	0.04*h*
Mesh-C	1	3,256,354	0.0016*h*	0.04h

### 3.2. Simulated tornado wind field

**Fig 5:**
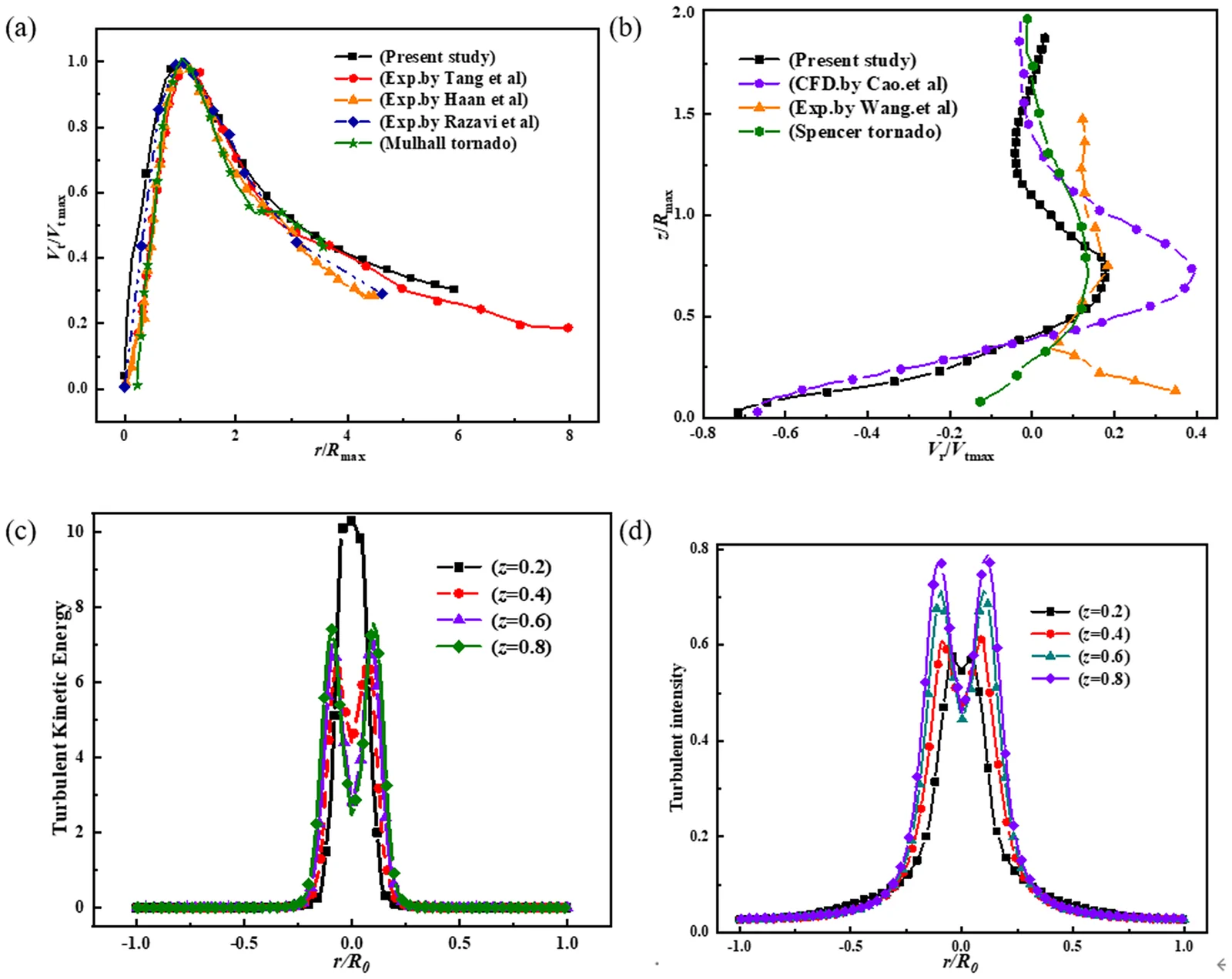
Tornado wind field characteristics, (a) radial distribution of tangential velocity, (b) radial velocity distribution, (c) radial distribution of turbulent kinetic energy, (d) radial distribution of turbulent intensity.

The tornado wind field is significantly influenced by tangential and radial velocities. The dependability of the simulation approach is examined in this study by fitting the simulation results to the measured Spencer tornado, and experimental data [31,36,37]. The tornado wind field characteristics are shown in Fig 5. The height near the ground is *z*. The tangential velocity within the core radius increases rapidly as the core radius develops, reaching its maximum value at the core radius point, as shown in Fig 5(a). The tangential velocity increases approximately linearly with the radius in the developing vortex core, and reaches the maximum value at the radius of the vortex core. Outside the radius of the vortex core, the velocity decays inversely proportional to the radius. According to Fig 5(b), it can be concluded that the radial velocity simulation results in this study are slightly different from the measured radial velocity of the Spencer tornado and the Wang’s experiment [36], and the radial velocity distribution of the simulation and experiment is consistent with the overall trend from outside to inside. A quantitative analysis reveals that the simulated tangential velocity in this study shows a Root Mean Square Error (RMSE) of 0.023 and a Mean Absolute Error (MAE) of 0.021 when compared to the experimental data by Tang. Furthermore, compared with the experimental data by Haan, the RMSE is 0.058 and the MAE is 0.057. These deviations are analyzed as part of the overall simulation uncertainty. The deviation is mainly because the measured tornado wind field is affected by various tangential velocity and roughness components near the ground, which makes the updraft of the tornado wind field change. Table 4 shows the error analysis of simulation parameters and experimental data.

**Table 4:** Error analysis of simulated parameters compared to experimental datasets.

Parameter	Dataset (Simulation vs. Reference)	Sampling Position	Error Measure (RMSE/ MAE)
Roof pressure Coefficient	Present CFD (Mesh-B) vs. TTU Exp. (Verma)	Center line AB on flat roof	RMSE:0.029/MAE:0.025
Tangential velocity	Present CFD vs. Tang Exp.	z= 0.5m	RMSE:0.023/MAE:0.021
Tangential velocity	Present CFD vs. Haan Exp.	z = 0.5m	RMSE:0.058/MAE:0.057
Radial velocity	Present CFD vs. Wang Exp	Core vertical section	RMSE:0.29/MAE:0.20

**Fig 6:**
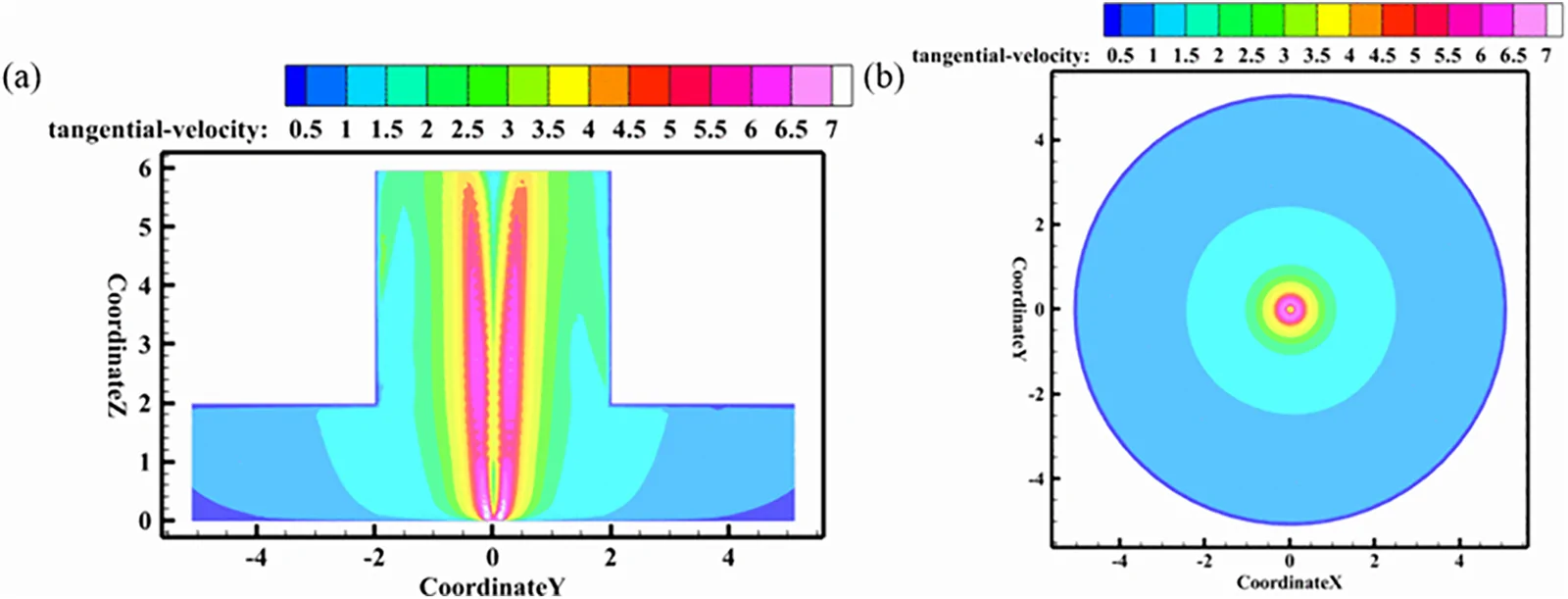
Tornado wind field profiles, (a) tangential velocity wind field vertical profile (x = 0m), (b) tangential velocity wind field horizontal profile (z = 0.5m).

Turbulence intensity is the most significant characteristic variable to characterize the properties of atmospheric turbulence. It indicates the relative intensity of fluctuating wind speed and describes the degree to which wind speed changes with time and space. The value of the more stable airflow away from the core is near to zero, and the turbulent kinetic energy and turbulent intensity are symmetrically distributed, as shown in Fig 5(c) and (d). The turbulent kinetic energy increases sharply near the core region and then decreases suddenly, and the turbulent kinetic energy and intensity in the core region increase steadily with the increase of the height *z* of the inflow region. The tornado wind field profile is given in Fig 6. The tangential velocity is shown in concentric circles with a symmetric distribution, as can be seen in Fig 6(a) and (b). The core radius’s tangential velocity reaches a maximum of 7m/s.

### 3.3. Ground roughness effects

The ground roughness was replicated by similar sand roughness in order to study the impact of ground roughness on wind pressure on hip roof. Ground roughness is set at the bottom of the tornado simulator by Blocken [38] using the logarithmic law of the atmospheric boundary layer (ABL) and the logarithmic law of the first-order continuous fitting of the wall function. The sand roughness height and roughness constant are denoted by *K*_s_ and *C*_s_, respectively, whereas *y*_0_ represents the aerodynamic roughness length. The numerical relationship between the aerodynamic roughness length *y*_0_ and the equivalent sand roughness height *K*_s_ is established to simulate the influence of surface roughness in tornado airflow. This was accomplished using the sand roughness height *K*_s_ and roughness constant *C*_s_ values of *K*_s_ = 20*y*_0_ and *C*_s_ = 0.5. When *y*_0_ = 0.03 m, *K*_s_ equals 0.6 in the lowest grass-covered plain scenario. For rough open terrain *y*_0_ = 0.1 m, then *K*_s_ = 2. *K*_s_ = 10 on extremely rough terrain when *y*_0_ = 0.5 m. If urban areas are considered, *K*_s_ = 40 when *y*_0_ = 2.0 m. Smooth ground is defined as having boundary conditions set to the ground so that, in the absence of ground friction, the roughness height *K*_s_ = 0 and the roughness length *y*_0_ = 0.

**Fig 7:**
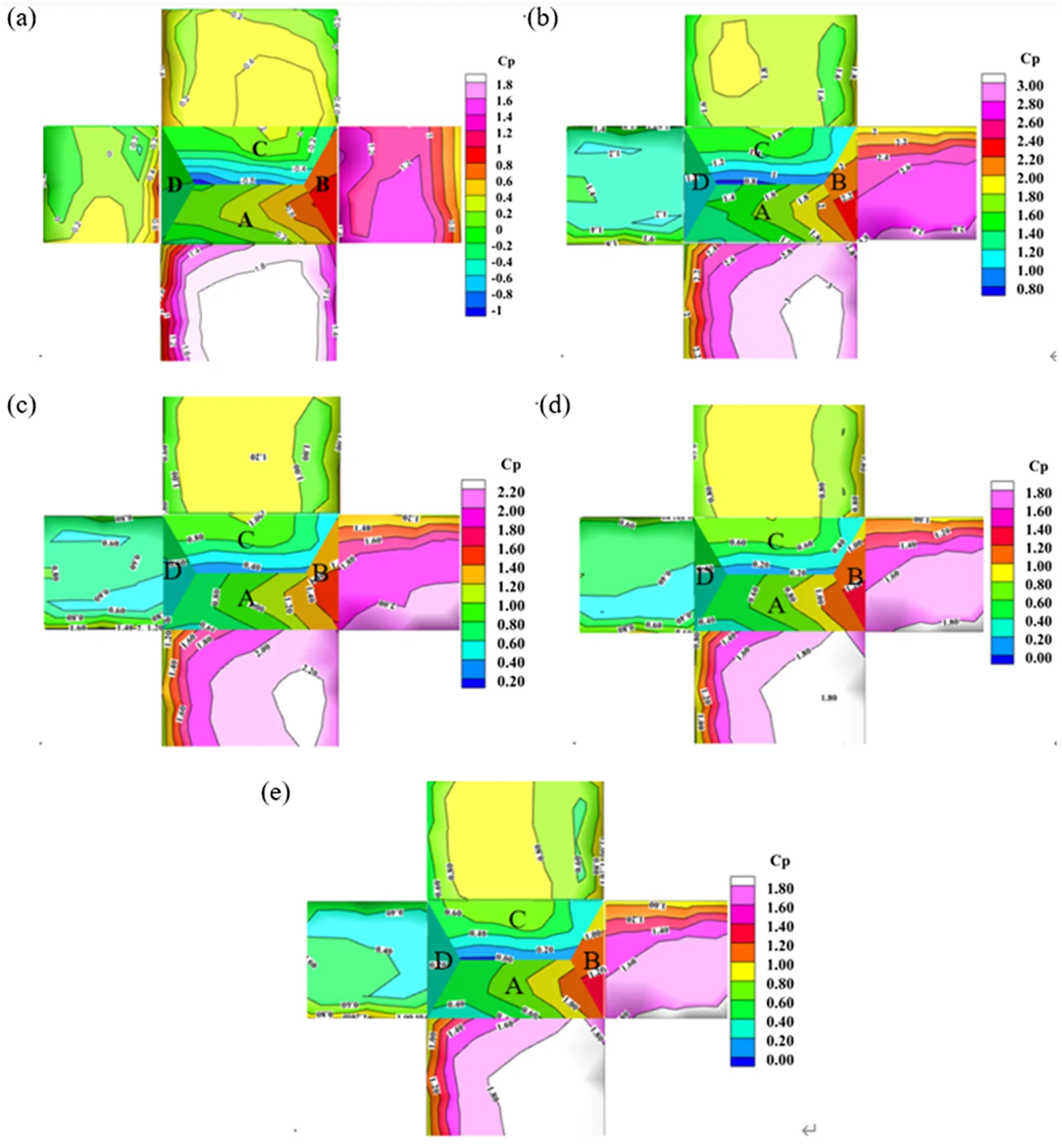
Wind pressure coefficients on the surface of building models with different ground roughness. (a) *K*_s_ = 0, (b) *K*_s_ = 0.6, (c) *K*_s_ = 2, (d) *K*_s_ = 10, (e) *K*_s_ = 40.

The deviation and disturbance of the core vortex occur as a result of the building model’s disruption of the core air flow at the core position. The air flow surrounding the building model is obstructed, air flow separates, a vortex forms, and the wind pressure coefficient varies significantly. The two aspects lead to the wind pressure coefficient on the surface of the building model is not symmetrical distribution. The wind pressure coefficients on the surface of the building model with various ground roughness are shown in Fig 7. The swirl ratio of the wind field is 0.18, and the low-rise building is located at the core of the wind field. The windward side C at the roof ridge displays negative pressure when the ground is smooth. Increased ground roughness acts as a physical friction barrier that slows the tangential velocity and intensifies the core updraft, thereby lowering the effective swirl ratio and shifting the roof pressure from negative to positive. When the roughness height *K*_s_ = 0.6, the wind pressure on the roof D surface reaches the maximum. The maximum wind pressure coefficient of 3.0 is also seen on the side wall of roof A, which is immediately impacted by the airflow. The pressure difference between the windward and leeward sides of smooth ground is larger due to the upward trend of the core airflow, while the pressure difference between the windward and leeward roof sides of rough ground is smaller. Leeward surface C is close to the ridge, due to the occurrence of airflow disturbance and separation, the formation of vortex, the smooth ground when the negative pressure, the rough ground is positive pressure, and the pressure is smaller. Compared with the smooth wall, the fluid flow on the rough ground overcomes the resistance of the rough element and converts more ordered kinetic energy into disordered turbulent kinetic energy.

**Fig 8:**
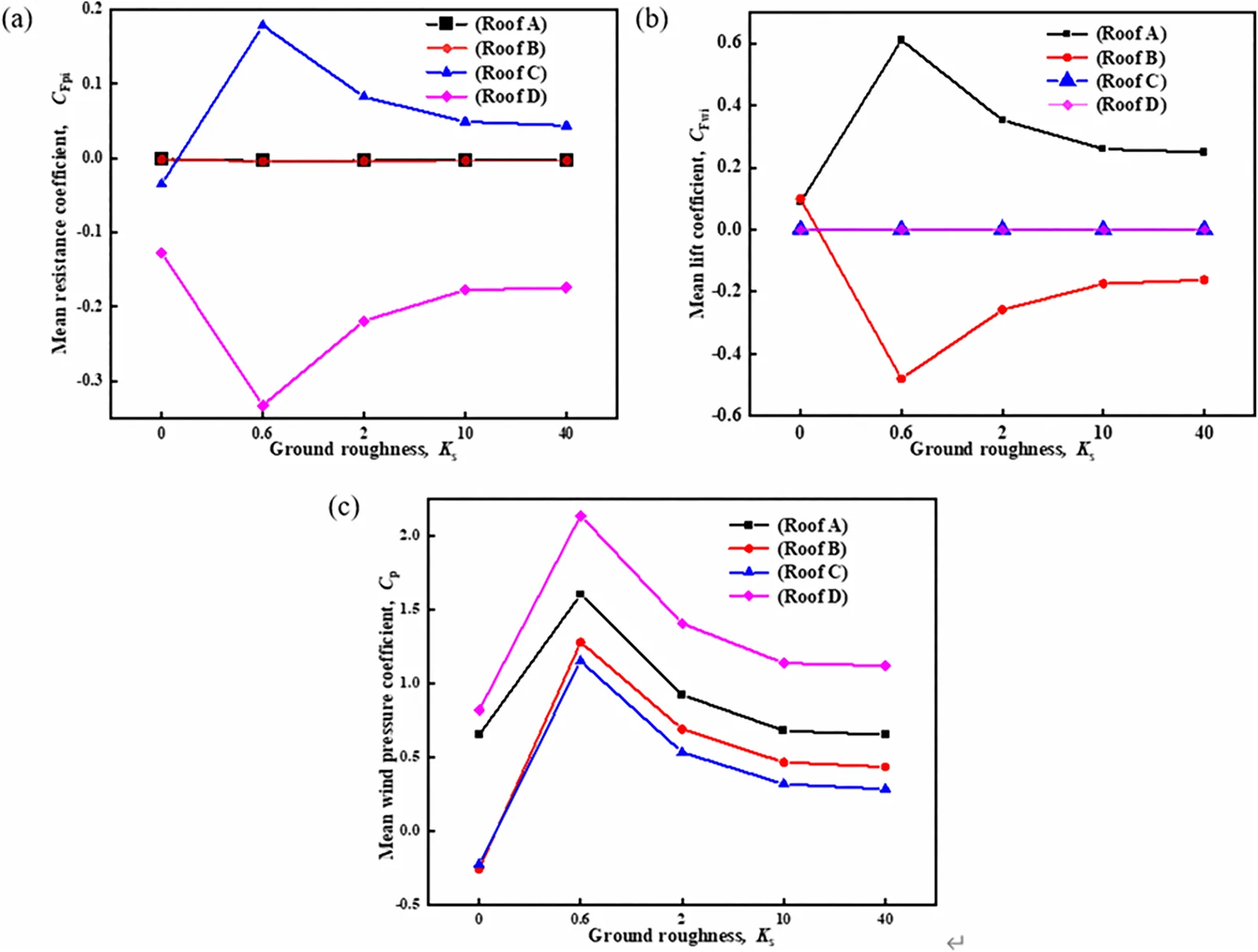
Hip roof with different ground roughness. (a) mean resistance coefficient, (b) mean lift coefficient, (c) mean wind pressure coefficient.

The effect of roughness on the aerodynamic characteristics of the building model surface is analyzed as shown in Fig 8. The lift curves of windward surface A and B drag and leeward surface C and D tend to horizontal values close to 0. Lift results from the load’s direct impact on the windward surface, while drag results from the leeward surface’s airflow separation and disturbance. The airflow is hindered by the ground’s roughness, which also causes the updraft in the core region to increase and the tangential velocity to drop, both of which have a similar impact of lowering the swirl ratio. This is consistent with the findings of Natarajan [39] and Zhang [40]. The lift resistance increases significantly at a roughness height of 0.6 on the windward side A and B and the leeward side C and D. The drag coefficient decreases with the increase of roughness height. The tangential velocity decreases more and the proportion of updraft increase decreases as a result of the greater rough height. Interestingly, the data demonstrates that at extremely high ground roughness levels, the severe airflow obstruction leads to a decline in the updraft intensity, causing the aerodynamic properties... to revert to trends similar to those observed in the smooth-ground wind field.

### 3.4. Swirl ratios effects

**Fig 9:**
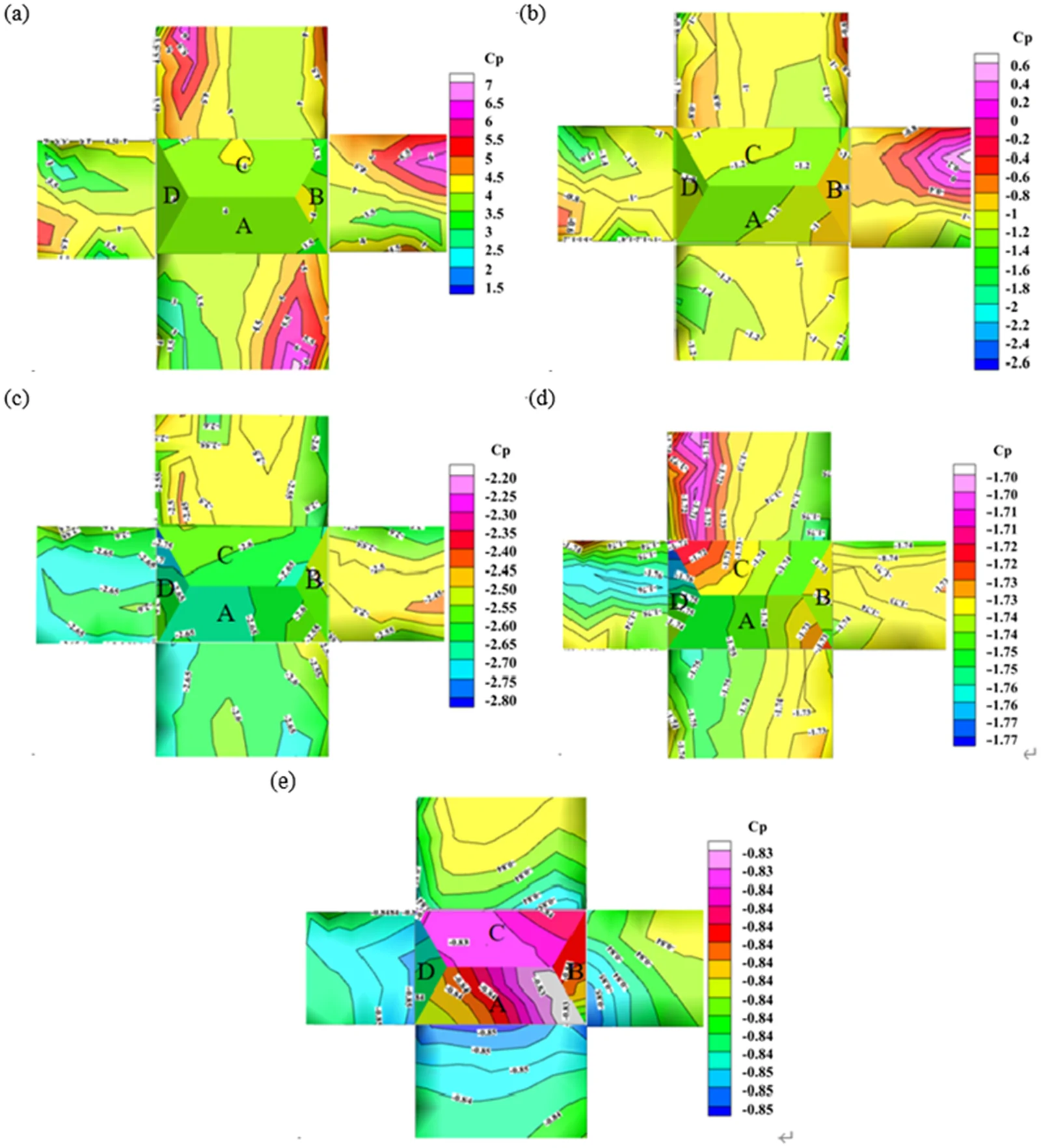
Wind pressure coefficients of hip roof with different swirl ratios. (a) *S*=0.1, (b) *S*=0.16 (c) *S*=0.3, (d) *S*=0.5, (e) *S*=0.72.

The inlet wind speed angle and the tornado wind field are significantly impacted by the swirl ratio. The wind pressure coefficients of hip roof at the center of the wind field, with swirl ratios of 0.1, 0.16, 0.3, 0.5, 0.72, and 0.8, are shown in Fig 9. The low-rise building is located in the core of the tornado wind field. The wind pressure coefficient of the roof changes dramatically as the swirl ratio rises. The airflow is updraft and the wind pressure coefficients on the roof are positive when the low swirl ratio is 0.1. The airflow at the core is downward at a high swirl ratio of 0.72, and the wind pressure on the roof is negative. The negative pressure on the roof is at its greatest when the swirl ratio is 0.3. Whenever the swirl ratio is between 0.3 and 0.72, the negative pressure value steadily decreases. The airflow disturbance and wind pressure of the wind field are significantly impacted by the vortex’s shift.

**Fig 10:**
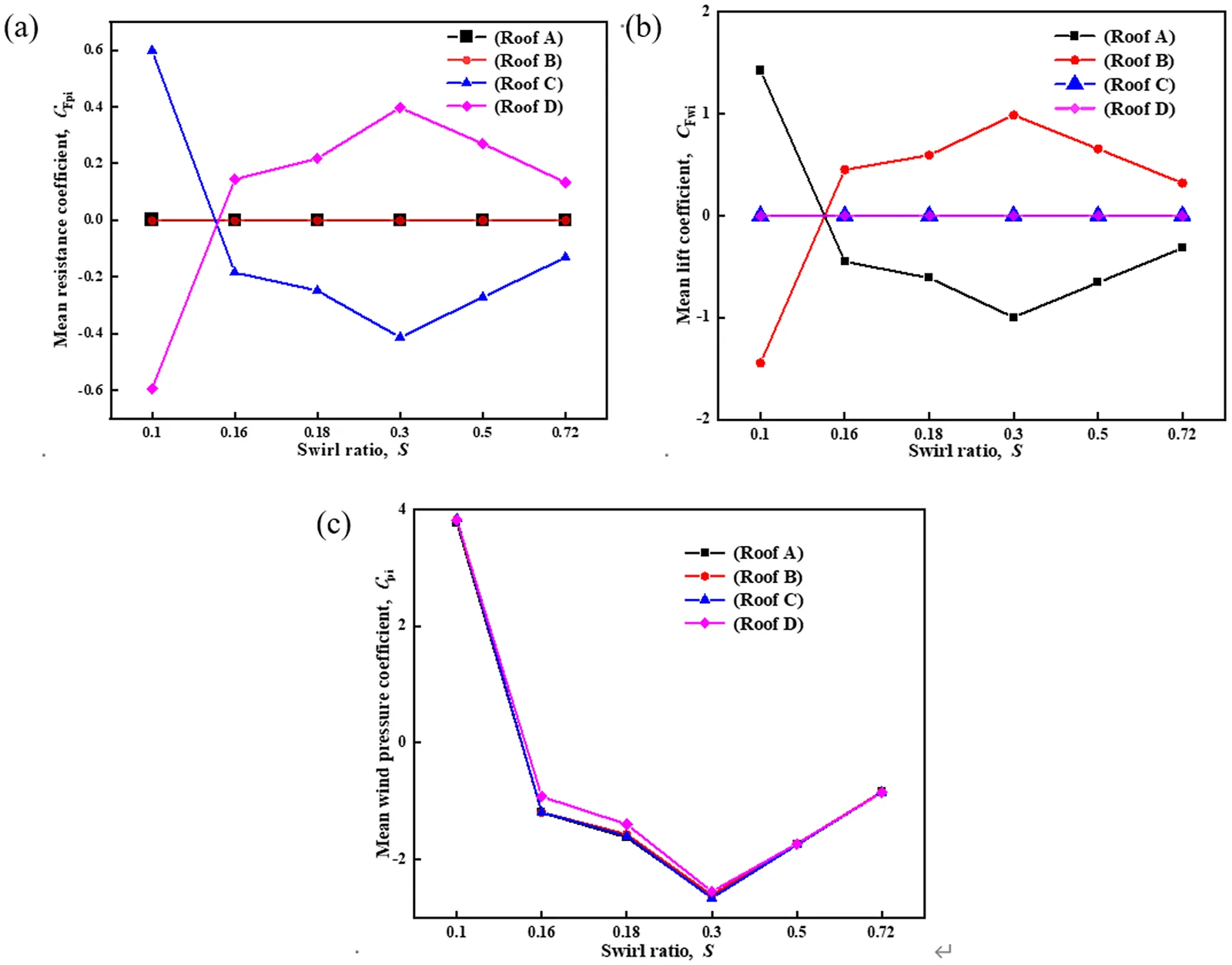
Hip roof with different swirl ratios, (a) mean resistance coefficient, (b) mean lift coefficient, (c) mean wind pressure coefficient.

The aerodynamic coefficients of roofs with different swirl ratios are given in Fig 10 Both the lift force on the leeward side and the drag force on the windward side rapidly rise when the swirl ratio approaches 0.1. The drag force on the leeward side C and D and the lift force on the windward side A and B both peak at the swirl ratio of 0.1. The airflow is updraft, the roof surface exhibits suction, and the lift force is at its highest due to the low swirl ratio. The absolute value of drag rapidly reduces as the swirl ratio rises to 0.16. The drag rapidly rises after the swirl ratio reaches 0.3. Finally, the resistance gradually reduces as the swirl ratio rises. Roof has a greater lift on the windward side and a greater drag on the leeward side with low swirl ratios and swirl ratios of 0.3. The wind field is mainly updraft and the roof surface is susceptible to lift, which is a greater positive pressure, when the swirl ratio is 0.1. The wind pressure on the roof surface rapidly falls as the swirl ratio rises, and as a result, the negative pressure steadily rises. The negative pressure reaches its peak at a swirl ratio of 0.3 and then steadily declines as the swirl ratio rises.

### 3.5. Locations effects

**Fig 11:**
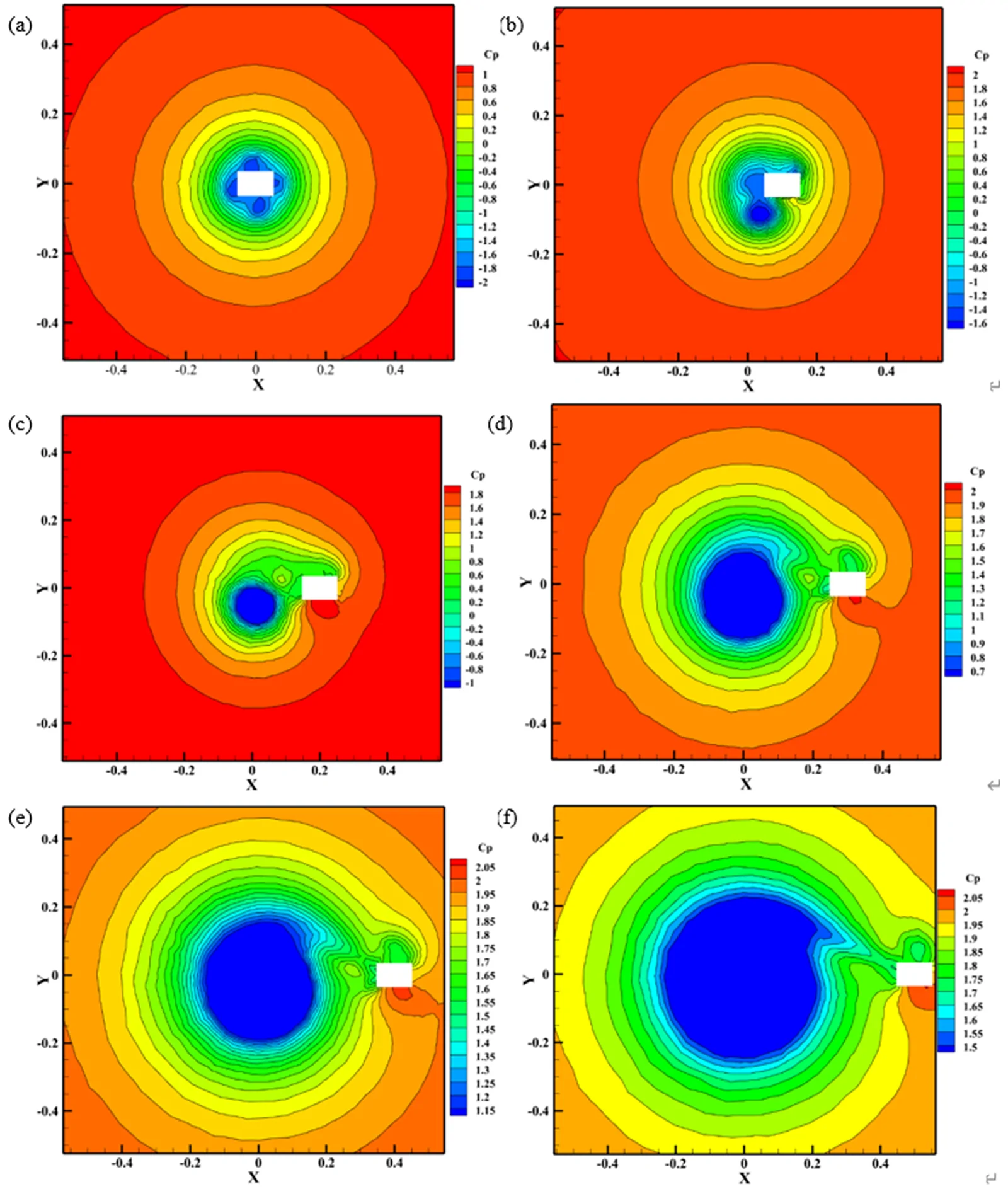
Distribution of wind pressure coefficients around the model height, (a) *r* = 0 *R*_max_, (b) *r* = 0.5*R*_max_, (c) *r* = 1.0*R*_max_, (d) *r* = 1.5*R*_max_. (e) *r* = 2.0*R*_max_, (f) *r* = 3.0*R*_max_.

To better demonstrate the variation in the wind field surrounding the model, Fig 11 shows the wind pressure distribution around the model at different positions using the building model’s height of z = 0.06m and the swirl ratio *S*=0.18. The vortex ratio of the wind field is 0.18, and the rotation angle of the low-rise building is 0°. The wind pressure surrounding the wind field is negative and symmetrically distributed when the model is situated in the center of the wind field. The size and location of the wind field core area change at *r* = 0.5*R*_max_, which has a significant impact on the wind pressure surrounding the model. The interaction between the model and the wind field is less, and the wind pressure all around the model is positive at *r* = 1.5*R*_max_. The interaction between the tornado and the building alters the properties of the tornado wind field as the model gets closer to the wind field core.

**Fig 12:**
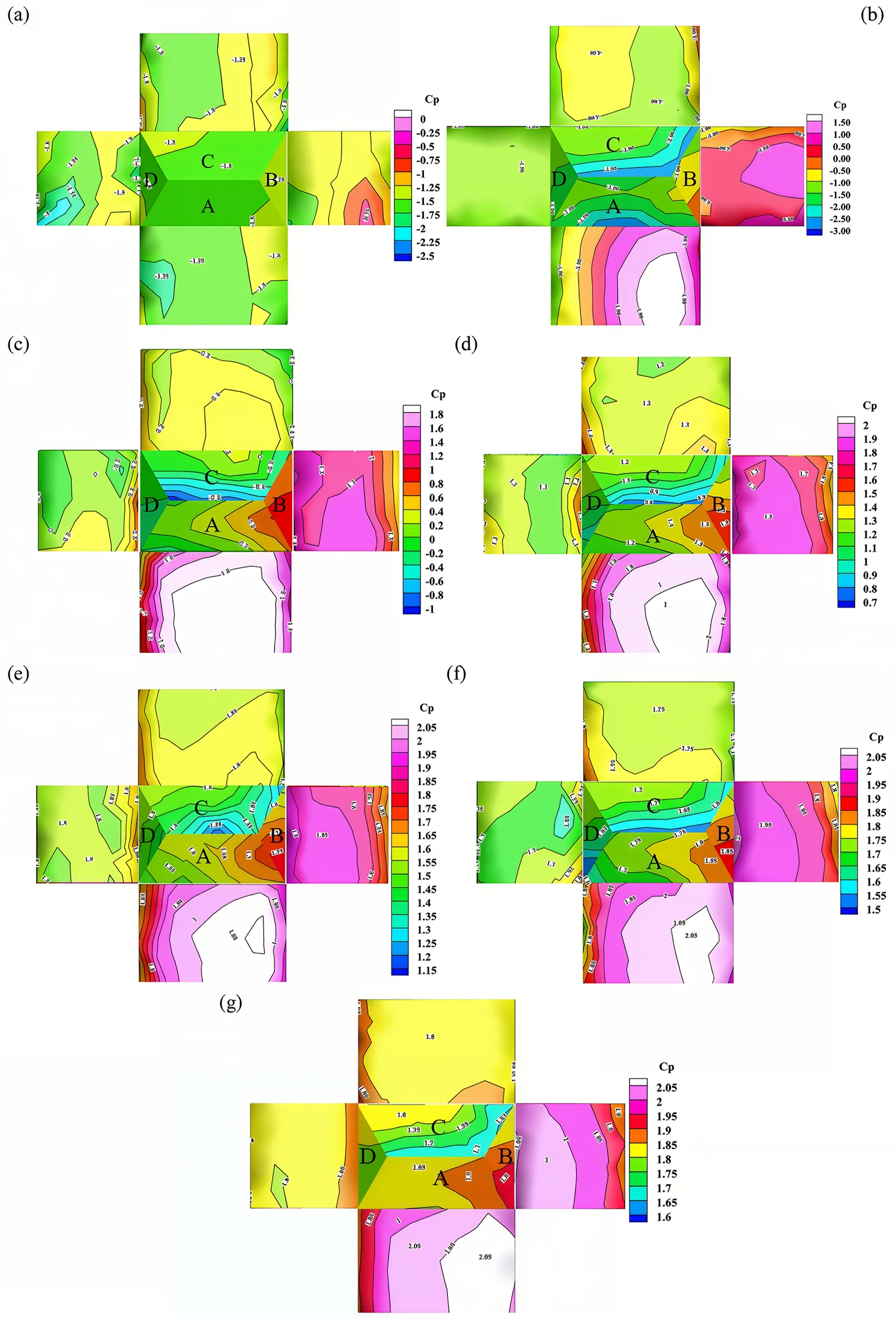
Wind pressure coefficients on the surface of the model building located at different locations in the wind field. (a) *r* = 0 *R*_max_, (b) *r* = 0.5*R*_max_, (c) *r* = 1.0*R*_max_, (d) *r* = 1.5*R*_max_, (e) *r* = 2.0*R*_max_, (f) *r* = 2.5*R*_max_, (g) *r* = 3.0*R*_max_.

The wind pressure coefficients on the surface of the building model placed at various places of the wind field are provided in Fig 12 to help analyze the impact of various locations on the wind pressure on the surface of the building model more effectively. The wind forces on the roof surface are all negative when *r* = 0*R*_max_. The greatest shift in the wind pressure distribution occurs at *r* = 0.5*R*_max_. A significant negative pressure, windward surface is located on the roof C and D ridge at the ridge and the wall. The positive and negative pressure between a ridge and a wall creates front pressure and rear suction, which makes the roof ridge vulnerable to damage. The wind pressure at the wall surface is positive and rising as it advances away from the wind field’s center when *r* ≧ 1.0*R*_max_. The difference between the pressure coefficients on the windward side walls and the leeward side walls in the core region is the biggest, and it gradually decreases between the pressure coefficients on the windward and leeward sides of the roof. There is a significant pressure drop in the core of the tornado, and the roof of the building model is subjected to a large vertical force, so when a tornado occurs, the building is destroyed and the roof of the model is most likely to lift.

**Fig 13:**
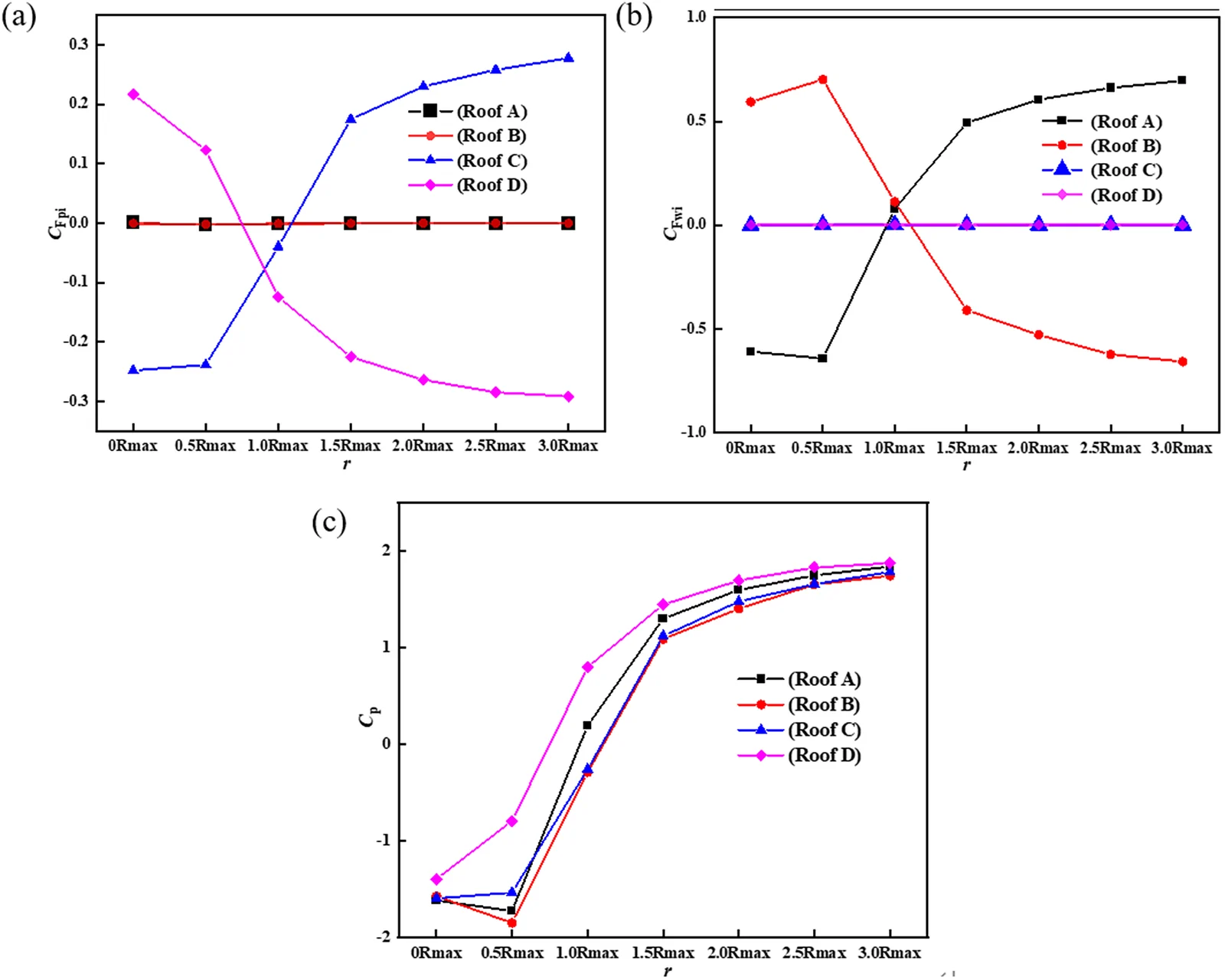
Hip roof at different locations, (a) mean resistance coefficient, (b) mean lift coefficient, (c) mean wind pressure coefficient.

The aerodynamic characteristics of the surface of the building model located at different locations in the wind field are given in Fig 13. Leeward surface lift and windward surface drag are very near to zero. Leeward surface drag and windward surface lift both have higher values. The drag forces on roofs C and D and the lift forces on roofs A and B show opposite trends as they move away from the core. The tornado vortex core damages the building model further, the turbulent updraft of air flow intensifies, and the lift on the model surface increases at *r* = 0.5*R*_max_, the point at which the lift on the windward side achieves its greatest value. The leeward surfaces C and D exhibit maximum resistance at the center of the wind field, which diminishes as one moves away from the center. The maximal negative pressure on the roof is present within the core radius and gradually decreases as one gets away from the core. The mean wind pressure is nearly zero when *r* = 1.0*R*_max_. The mean wind pressure on the roof surface increases quickly outside the core radius before gradually settling. The highest roof B negative pressure value is at *r* = 0.5*R*_max_, and it steadily increases away from the core as the positive pressure value.

### 3.6. Building orientations effects

**Fig 14:**
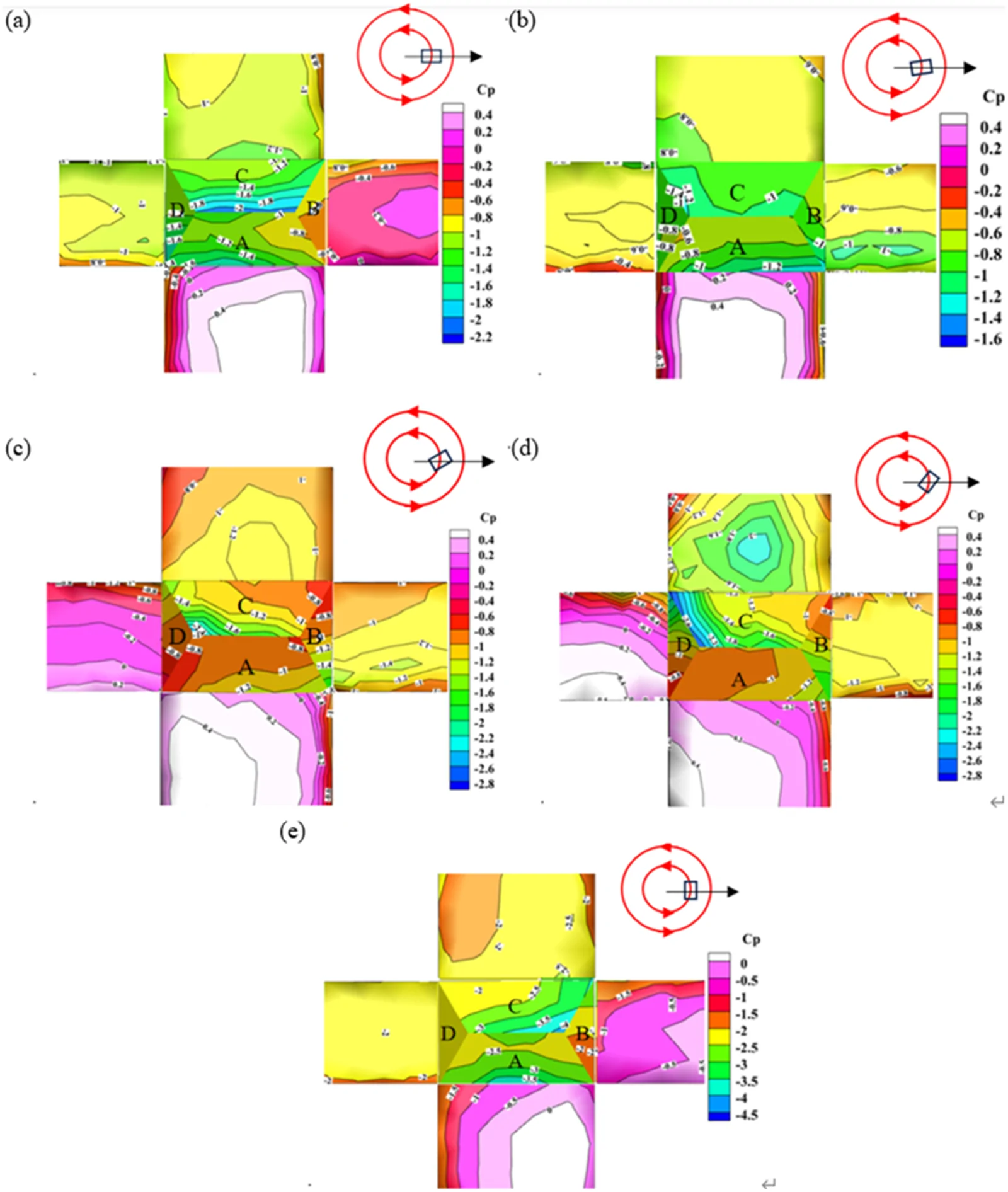
Different orientations of the building model at the maximum core radius (a) *β* = 0°, (b) *β* = 30°, (c) *β* = 45°, (d) *β* = 60°, (e) *β* = 90°.

As shown in Fig 14, the wind pressure coefficients on the surface of the building model with a swirl ratio of 0.5 and building orientation of 0°, 30°, 40°, 60°, and 90° at the maximum core radius are provided in this study. The wind pressure lines on both sides of the wall of roof A are dense and the wind pressure distribution substantially alters when the model’s building orientation is 30°. Because of the separation bubble and vortex created by the airflow separation, the negative wind pressure coefficient at the ridge where roofs C and D meet achieves its maximum value of −1.4. The airflow divides and disrupts at the crest of roofs C and D when the building orientation is 60°, creating a vortex and exhibiting the greatest negative pressure coefficient of −2.6. The airflow is less constrained and moves faster when the building orientation is 90° since the windward side is now the D side. The overall negative pressure on the surface of the building model increases to its maximum due to an increase in the pressure drop and pressure difference of the air. The maximum negative pressure coefficient at the ridge of roof C near A and B is −4. The maximum negative pressure is seen at the intersection of the ridge on the leeward side due to airflow separation. The negative pressure on the roof surface of the building model is greatest and most vulnerable to damage at the building orientation 90°, when the windward area is smallest.

**Fig 15:**
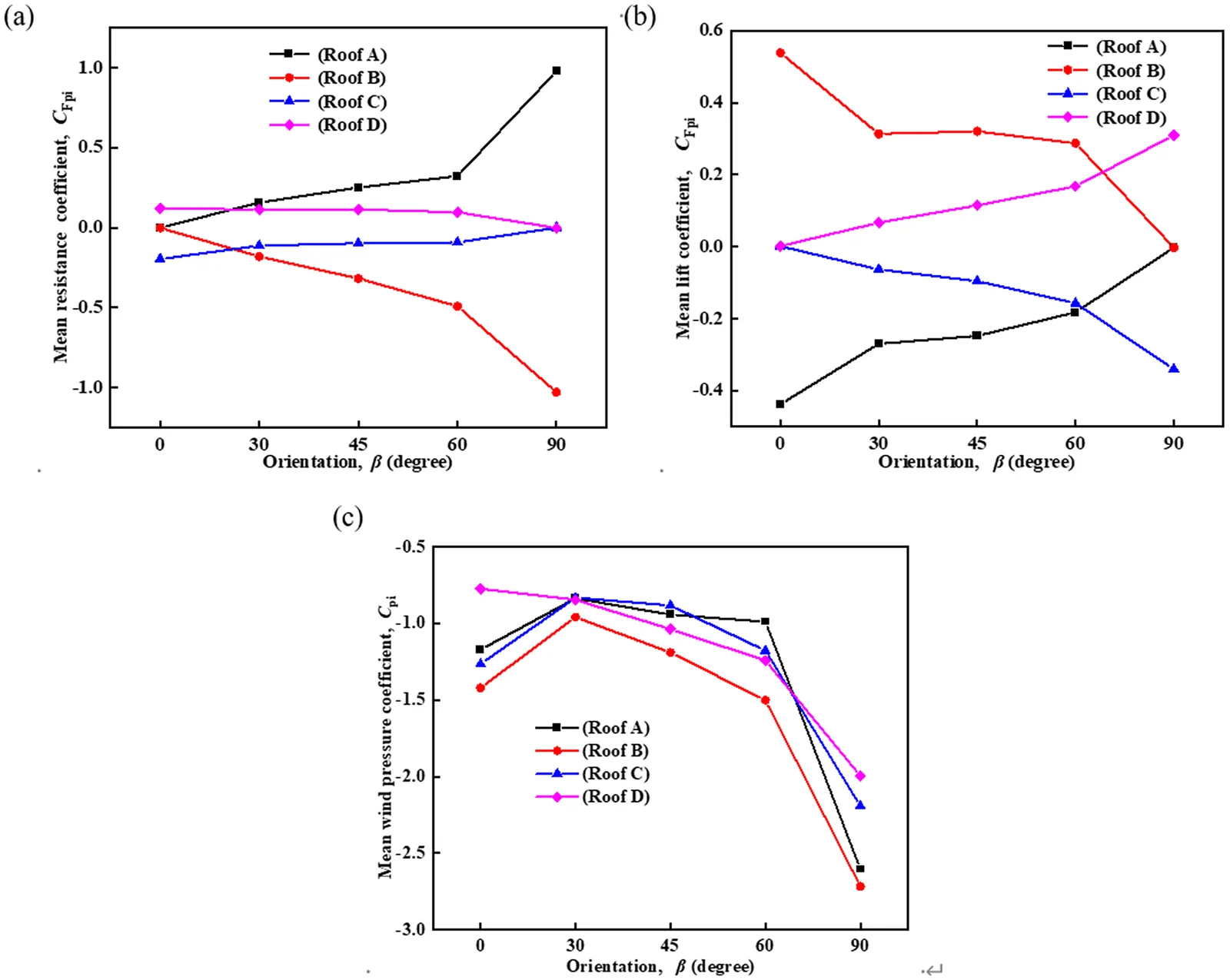
Aerodynamic coefficients of hip roof with different orientations (a) mean resistance coefficient, (b) mean lift coefficient, (c) mean wind pressure coefficient.

The aerodynamic force coefficients of hip roof with various building orientations are shown in Fig 15 in order to more thoroughly explore the impact of building orientation on the aerodynamic force of the building model surface. The mean drag coefficient of the model roof is relatively stable from 0° to 60° of the building orientations. The mean drag coefficients of roofs A and B first rise gradually with the building orientation, but at a 90° building orientations, the drag spikes and achieves its highest value. The windward area diminishes, the hindrance to the surrounding airflow lowers, the airflow speed increases, the pressure drop and resistance increase because the windward surface switches from A to D as the building model’s tilt approaches 90°. Roofs A and B achieve their greatest lift at an angle of 0°, and as the angle increases, the mean lift coefficient gradually declines. The lift force grows when the airflow disruption and separation of roof A occur more frequently, increasing the suction force. The mean lift coefficient of roofs C and D is close to 0 when the building orientations is 0°, and gradually increases as the building orientations of the building model increases. The surrounding air velocity and pressure drop increase as roofs C and D gradually shift from the leeward side to the windward side and from close to the core end to far from the core end, increasing the lift. The negative pressure of roofs A, B, and C gradually drops to 30° as the building model’s angle increases before abruptly increasing. The intersection of roofs A and D is confronting the effect of airflow at an angle of 30°, and the airflow separation is severe. The more direct airflow effect there is on the windward side of the roof D, the more negative pressure there is on the roof D. The roof’s negative pressure is at its highest at the 90° building orientation. This occurs when the windward area of the windward side D decreases, the airflow blockage lowers, the peripheral airflow speed increases, and the gap between airflow disturbances increases.

### 3.7. Wind field vortex around the building model in different locations

**Fig 16:**
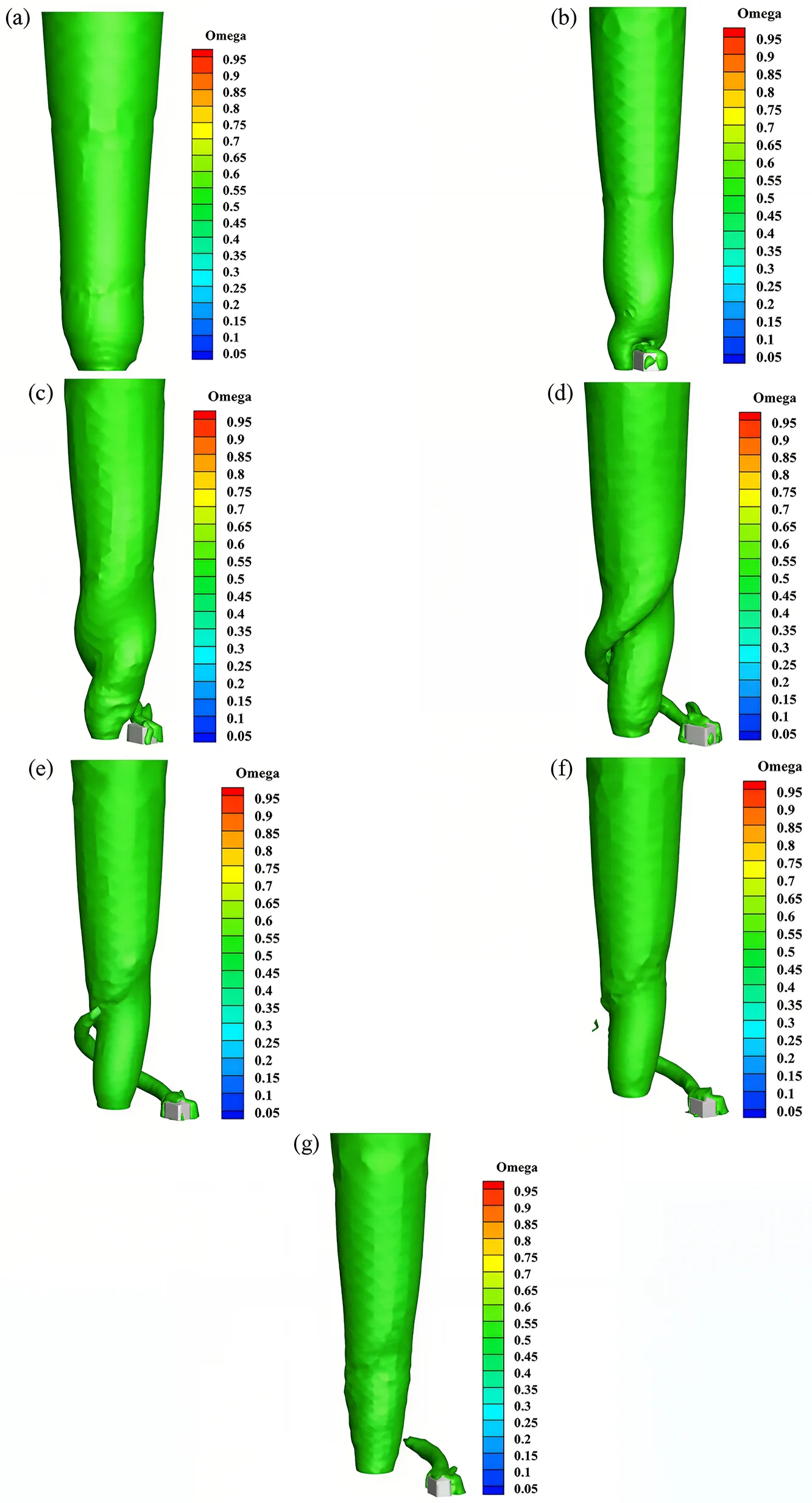
Distribution of vorticity around the building model at different wind field locations. (a) *r* = 0*R*_max_, (b) *r* = 0.5*R*_max_, (c) *r* = 1.0*R*_max_, (d) *r* = 1.5*R*_max_, (e) *r* = 2.0*R*_max_, (f) *r* = 2.5*R*_max_, (g) *r* = 3.0*R*_max_.

A new omega vortex identification technique [41] is applied, which is not affected by the threshold and captures both strong and weak vortices. The ratio of the vorticity in the vortex core to the total vorticity is represented by *Ω*, which is based on splitting the vorticity into the vortex component and the non-vortex part.Through a dimensionless parameter *Ω*, the rotational motion and non-rotational shear motion in the split flow field are effectively zoned, and the vortex structure is more accurately identified. The thresholds are normalized to 0–1, and a value of *Ω* = 0.52 is adopted to isolate the coherent vortex structures. The swirl ratio of 0.18 is selected for the various tornado wind field vortex sites illustrated in Fig 16. This specific value is well-established in the literature for the Omega method and is derived from the analysis of a linear boundary layer flow, where it accurately marks the transition point between rotational vortex dominance and shear dominance [15]. Utilizing this standardized threshold enhances the objectivity and reproducibility of our vortex identification process, allowing for a consistent and accurate analysis of both strong vortices in the tornado core and weaker vortices generated around the hip roof geometry.

The tornado core vortex totally envelops the modelled model while it is in the core position. The model began to separate from the main vortex at *r* = 0.5*R*_max_, and the main vortex was severely disrupted by the building model. The model established a large number of vortices on the leeward side. The wall of windward side A exhibits zero vortex volume at *r* = 1.0*R*_max_, while the wall of leeward side exhibits a significant quantity of vortex volume. Pronounced vortices are present on the roof’s leeward side, resulting from airflow separation initiated by the direct impact on the windward side. From the leeward side of the model, the branch vortex begins to grow. A swirling upward branch vortex is stretched from the intersection of C and D on the leeward side of the building model to join with the main vortex when *r* ≧ 1.5*R*_max_, as the model travels away from the tornado core. The branch vortex generated by the model is separated from the main vortex at *r* = 3.0*R*_max_. The core vortex of the wind field has little effect on the building model because the building model is far away from the core of the wind field.

## 4. Conclusion

The interaction between the tornado wind field and the building model was investigated using the SST k−ω turbulence model. The wind load characteristics of the hip roof during tornado action were explored based on the numerical model of the Ward-type tornado wind simulator. The following conclusions were obtained:

(1) The emergence of ground roughness hinders the near-surface airflow, reduces the airflow velocity, increases the updraft, and increases the lift of the roof due to the increase of the surface suction of the building model. This finding confirms the experimental results of Razavi [42]. Low ground roughness causes the airflow to be obstructed, the tangential velocity to drop, the updraft to grow, and this in turn raises the suction force on the roof surface. When the ground roughness is high, the amplitude of the updraft decreases, and the aerodynamic characteristics similar to the smooth ground wind field. When selecting sites for significant structures in low-roughness zones, the wind-resistance design of their roofs must account for more severe wind suction forces.
(2) The wind field becomes updraft and the roof surface is raised when the swirl ratio reaches 0.1. The largest negative pressure is present at the intersection of the leeward roof ridge when the building orientations is 90° because of the airflow separation and the minimal windward region. As a result, the model is easily destroyed. In structural design, particular emphasis should be placed on reinforcing the ridge area, especially the connection nodes on the leeward ridge.
(3) The largest negative pressure and most significant shift in the wind pressure distribution on the roof occur at *r* = 0.5*R*_max_. The ridge of the leeward side C shows negative pressure, while the ridge of the windward side A shows positive pressure, and the positive and negative pressures cause the ridge of the roof to be easily damaged. The building model on the tornado’s vortex nucleus damage is greater, the air flow turbulence updraft rose, and the lifting power on the windward side was at its greatest.
(4) The wall of A displays no vortex volume on the windward side when *r* = 1.0*R*_max_, but a significant amount of vortex volume on the leeward side. When the four-slope roof is designed to resist tornado, the roof ridge and the orientation of the side wall of the four-slope roof are greatly affected and should be strengthened. Attention should be focused on the case of the maximum core radius and the orientation 90°, when low-rise buildings are most vulnerable to damage. Table 5 shows the main contributions and progress of this study in this field.

**Table 5:** Summary of the study’s key contributions and advancements to the field.

Key Investigated Aspects	Main Contributions	How It Advances the Field
Ground Roughness	Identified non-linear effects of roughness: low roughness increases updraft and roof surface suction, while high roughness produces aerodynamic characteristics similar to a smooth wind field.	Advances site-specific structural design guidelines. Specifically warns that buildings in low-roughness zones require enhanced wind-resistance design to withstand more severe suction forces.
Swirl Ratio & Building Orientation	Revealed that at a swirl ratio of 0.1 and a 90° orientation, the maximum negative pressure is concentrated at the intersection of the leeward roof ridge due to airflow separation.	Pinpoints exact structural vulnerabilities under tornado loads, directing engineers to prioritize the reinforcement of leeward ridge connection nodes in low-rise building designs.
Vortex Core Radius (*r* = 0.5*R*_max_)	Demonstrated that the largest negative pressure, maximum lifting power, and most significant pressure distribution shifts occur exactly at *r* = 0.5*R*_max_.	Clarifies the localized aerodynamic mechanisms of extreme roof damage, highlighting the critical zone within the tornado path where buildings experience maximum destructive lifting forces.
Outer Radius (*r* = 1.0*R*_max_)	Mapped the vortex volume distribution, showing significant vortex generation on the leeward wall at *r* = 1.0*R*_max_, making hip roofs highly susceptible to damage.	Provides targeted strengthening strategies for hip roofs, emphasizing the necessity of reinforcing roof ridges and side-wall orientations when subjected to the maximum core radius.

### 4.1. Limitations and future work

Employing a single Ward-type generator with a model at a geometric scale of 1:100 does indeed impose limitations in several respects. Firstly, challenges arise concerning fluid dynamic similarity. The ratio of tangential to radial velocities within the model typically falls far below that observed in actual tornadoes. Secondly, the scaled model exhibits Reynolds number discrepancies compared to the actual tornado field. This leads to simulation deviations in detailed features such as vortex core structure and turbulence intensity, which restricts the direct extrapolation to full-scale building loads, as model-scale simulations at lower Reynolds numbers typically underestimate the localized extreme suctions on roof ridges and corners. Despite these constraints, we employed the following methodologies in numerical simulations and result analysis to enhance credibility. Firstly, all measured data were expressed using dimensionless coefficients, reducing direct dependence on geometric scales. Finally, we compared experimental findings with observational data from actual tornado fields and high-resolution numerical simulations, revealing qualitative consistency in primary vortex structures and load trends.

This study examines only parameters such as vortex ratio and ground roughness. It does not consider the impact of higher-intensity tornadoes (EF4-EF5) on low-rise buildings. Furthermore, future research will incorporate fluid-structure interaction techniques to conduct in-depth analyses of the structural response and potential fatigue damage to roofs and fasteners under transient tornado loads.

## Supporting information

S1 File Comparison and verification. (XLSX)

S2 File The influence of different ground roughness on Hip Roof. (XLSX)

S3 File The influence of different rotation angles on Hip Roof. (XLSX)

S4 File The influence of different spacing on Hip Roof. (XLSX)

S5 File The influence of different swirl ratios on Hip Roof. (XLSX)
